# Association between Human Prothrombin Variant (T165M) and Kidney Stone Disease

**DOI:** 10.1371/journal.pone.0045533

**Published:** 2012-09-19

**Authors:** Nanyawan Rungroj, Nirinya Sudtachat, Choochai Nettuwakul, Nunghathai Sawasdee, Oranud Praditsap, Prapaporn Jungtrakoon, Suchai Sritippayawan, Duangporn Chuawattana, Sombat Borvornpadungkitti, Chagkrapan Predanon, Wattanachai Susaengrat, Pa-thai Yenchitsomanus

**Affiliations:** 1 Division of Molecular Medicine, Department of Research and Development, Faculty of Medicine Siriraj Hospital, Mahidol University, Bangkok, Thailand; 2 Division of Molecular Genetics, Department of Research and Development, Faculty of Medicine Siriraj Hospital, Mahidol University, Bangkok, Thailand; 3 Medical Biotechnology Unit, National Center for Genetic Engineering and Biotechnology (BIOTEC), National Science and Technology Development Agency (NSTDA), Bangkok, Thailand; 4 Division of Nephrology, Department of Medicine, Faculty of Medicine Siriraj Hospital, Mahidol University, Bangkok, Thailand; 5 Khon Kaen Hospital, Khon Kaen, Thailand; Central China Normal University, China

## Abstract

We previously reported the association between *prothrombin* (*F2*), encoding a stone inhibitor protein - urinary prothrombin fragment 1 (UPTF1), and the risk of kidney stone disease in Northeastern Thai patients. To identify specific *F2* variation responsible for the kidney stone risk, we conducted sequencing analysis of this gene in a group of the patients with kidney stone disease. Five intronic SNPs (rs2070850, rs2070852, rs1799867, rs2282687, and rs3136516) and one exonic non-synonymous single nucleotide polymorphism (nsSNP; rs5896) were found. The five intronic SNPs have no functional change as predicted by computer programs while the nsSNP rs5896 (c.494 C>T) located in exon 6 results in a substitution of threonine (T) by methionine (M) at the position 165 (T165M). The nsSNP rs5896 was subsequently genotyped in 209 patients and 216 control subjects. Genotypic and allelic frequencies of this nsSNP were analyzed for their association with kidney stone disease. The frequency of CC genotype of rs5896 was significantly lower in the patient group (13.4%) than that in the control group (22.2%) (*P = *0.017, OR 0.54, 95% CI 0.32–0.90), and the frequency of C allele was significantly lower in the patient group (36.1%) than that in the control group (45.6%) (*P = *0.005, OR 0.68, 95% CI 0.51–0.89). The significant differences of genotype and allele frequencies were maintained only in the female group (*P = *0.033 and 0.003, respectively). The effect of amino-acid change on UPTF1 structure was also examined by homologous modeling and *in silico* mutagenesis. T165 is conserved and T165M substitution will affect hydrogen bond formation with E180. In conclusion, our results indicate that prothrombin variant (T165M) is associated with kidney stone risk in the Northeastern Thai female patients.

## Introduction

Kidney stone disease (KSD) is an important public health problem in the Northeastern (NE) population of Thailand [1], [2]. The etiology of KSD in this population is unknown; however, the disease seems to be different from what was reported in the Western and other ethnic groups because it is not associated with the conditions of increased urinary solutes such as hypercalciuria, hyperoxaluria, and hyperuricosuria [3], [4]. Previously, our group has reported evidence suggesting a genetic contribution to kidney stone disease in the NE Thai population since the disease has characteristics of familial aggregation with a high relative risk (λ_R_ = 3.18) among members of the affected families [5]. Defects of urinary stone-inhibitor proteins, which were found as matrix proteins in human kidney stone and shown to influence the formation of calcium-containing kidney stone, have been proposed to be involved in kidney stone formation [6], [7]. These proteins can inhibit kidney stone formation at different stages such as crystal nucleation, growth, aggregation, and binding to renal epithelial cells [8], [9]. In our recent work, we conducted an association study by genotyping 67 SNPs distributed within and flanking 8 candidate genes including *TFF1*, *S100A8*, *S100A9*, *S100A12*, *AMBP*, *SPP1*, *UMOD*, and *F2*, encoding trefoil factor 1, calgranulins (A, B, and C), bikunin, osteopontin, Tamm-Horsfall protein, and urinary prothrombin fragment 1, respectively. We firstly reported the association between *prothrombin (F2)* haplotype and KSD in the NE Thai female patients [10].

Human *prothrombin* (*F2*) (GenBank NM_000506, NP_000497) controls synthesis of prothrombin – the precursor of thrombin, also known as coagulation factor II (F2), a serine-protease coagulation protein in the blood stream that converts soluble fibrinogen into insoluble strands of fibrin, and catalyzing many other coagulation-related reactions. *F2*, located on 11p11-q12 and encompassing 20.3 kilobases (kb), encodes a protein consisting of 622 amino acids [11], [12]. Urinary prothrombin fragment 1 (UPTF1) is an F1 activation peptide of human prothrombin [13]. This 31-kDa glycoprotein was initially described as crystal matrix protein that was found to be the major protein incorporated within calcium oxalate (CaOx) crystals generated from human urine *in vitro* [14]. Amino acid analysis revealed that this protein contains γ-carboxyglutamic acid (Gla) residues located near its N-terminus, which are responsible for the calcium-binding activity of the protein [15]. It is a potent inhibitor of CaOx crystal growth and aggregation in undiluted human urine and under inorganic conditions [16], [17].

In the present work, we sequenced entire coding regions of *F2* to identify specific variation associated with KSD in the NE Thai patients. We have now reported the association between prothrombin variant, a substitution of threonine (T) by methionine (M) at the position 165 (T165M), and KSD in the NE Thai female patients.

## Materials and Methods

### Patients and Control Subjects

Two hundreds and sixteen patients with KSD (135 females and 81 males; aged 22–80 years) recruited from Khon Kaen Regional Hospital, in the northeastern part of Thailand, during 2004–2006 were studied. The controls were healthy people consisting of 216 age-matched unrelated individuals (126 females and 90 males; aged 22–84 years) who had no history of KSD and were also recruited from the same geographic area of patients. The details of the patients and control subjects have been described in our previous reports [5], [10]. Clinical characteristics of the 216 patients are presented in Table S1.

Diagnosis of KSD was performed by radiography of kidney-ureter-bladder (KUB), the scar and history of stone removal surgery, and in some suspicious cases by additional ultrasonography. The exclusion criteria of subjects were the presence of kidney stone secondary to all known causes (including renal tubular acidosis, primary hyperparathyroidism, inflammatory bowel disease, Cushing disease, hyperthyroidism, and drug-induced kidney stone) diagnosed by clinical history and symptoms, physical and laboratory, acute acid loading test, and serum electrolytes. Urine and blood sample were collected for electrolyte analyses. Stones after removal from the patients were analyzed by using Nicolet™ 380 Fourier Transform Infrared Spectrometer. The analysis of surgically removed stones from 86 patients showed that the stones from 79 patients (92%) contained calcium salts (whewellite, dahllite, and weddellite) and the stones from remaining 7 patients (8%) comprised of uric acid and struvite (Table S1). Thus, these 7 patients with non-calcium stones were excluded and the remaining 209 patients (132 females and 77 males) were subject to further study.

The study project was approved by Siriraj Institutional Review Board and the Ethics Committee of the Ministry of Public Health, Thailand. Informed consent was signed by the subjects before study. Genomic DNA samples from the patients and control subjects were extracted from their peripheral blood samples by using standard phenol-chloroform method.

### Sequencing of *F2*

Specific primers for PCR amplifications and sequencing of the promoter region, all 14 exons, and exon-intron boundaries of *F2*, were designed by Primer3 program (http://frodo.wi.mit.edu/primer3/) and listed in Table S2. The PCR was performed by using the IMMOLASE™ DNA polymerase (Bioline, USA) with 0.5 pmol each of forward and reverse primers, 1x Taq polymerase buffer, 2–3 mM MgCl_2_ and 100 ng of genomic DNA sample. The cycling condition was set as follows: initial incubation step at 94°C for 10 minutes, 30 cycles of denaturation at 94°C for 30 seconds, annealing at 68°C for 1 minute (for promoter 1 and exons 3 and 4) or 66°C for 45 seconds (for exons 7 and 8) or 64°C for 45 seconds (for promoter 2, exons 1 and 2, and exons 5 and 6), and extension at 72°C for 45 seconds (excepting for the cycling of promoter 1, extension at 72°C for 90 seconds was used). After the final step at 72°C for 10 minutes, the PCR products were examined by agarose gel electrophoresis. The PCR profile and condition were adjusted until a single discrete band was obtained. The PCR products were treated with ExoSAP-IT® (Affymetrix, USA) before processing for direct sequencing. PCR sequencing reaction was conducted under BigDye™ Terminator Cycling conditions and analyzed by 3730XL DNA analyzer (Applied Biosystems™, USA) by a service provider – Macrogen Inc (South Korea). The sequencing data were analyzed by comparing with the reference nucleotide sequence of *F2* (NC_000011.9) by multiple sequence alignment using ClustalW2 program (http://www.ebi.ac.uk/Tools/clustalw2/index.html).

### Genotyping of Single Nucleotide Polymorphism

The genotype of a single nucleotide polymorphism (SNP rs5896) was carried out by the polymerase chain reaction-restriction fragment polymorphism (PCR-RFLP) method. The 733-bp PCR fragment containing rs5896 was amplified by using F2F4 and F2LR1 as forward and reverse primers, respectively (Table S2). An initial PCR denaturation step was performed at 94°C for 10 minutes, followed by 30 cycles of denaturation at 94°C for 30 seconds, annealing at 64°C for 45 seconds and extension at 72°C for 45 seconds, with a final 10-minute extension at 72°C. The PCR product was digested with 10 U of *Nco*I (BioLab, England) at 37°C overnight. The digested products were subjected to electrophoresis on 3% agarose gel and the results were recorded and evaluated.

### Structural Analysis of Human Prothrombin Fragment 1

The three-dimensional (3D) structures of both wild-type and variant human prothrombin fragment 1 were constructed by homology modeling. The most suitable template was initially determined by alignment of target sequence and template structure and the one with highly identity value alignment was selected. The model was built and evaluated for quality by using the SWISS-MODEL workspace (http://swissmodel.expasy.org/workspace/). Alteration of H-bond forming pattern caused by variant protein was examined by using PyMOL VERSION 0.99rc6 (DeLano Scientific LLC, Palo Alto, California, U.S.A.).

### Statistical Analysis

Statistical analysis for Hardy-Weinberg equilibrium (HWE), association between SNP genotype, allele, or haplotype frequencies and disease phenotype were performed by SNPstats program (http://bioinfo.inconcologia.net/snpstats/start.htm) and Haploview 4.1 software. *P* value <0.05 was considered statistically significant in the comparisons of their differences.

## Results

### Sequencing of *F2*

**Figure 1:**
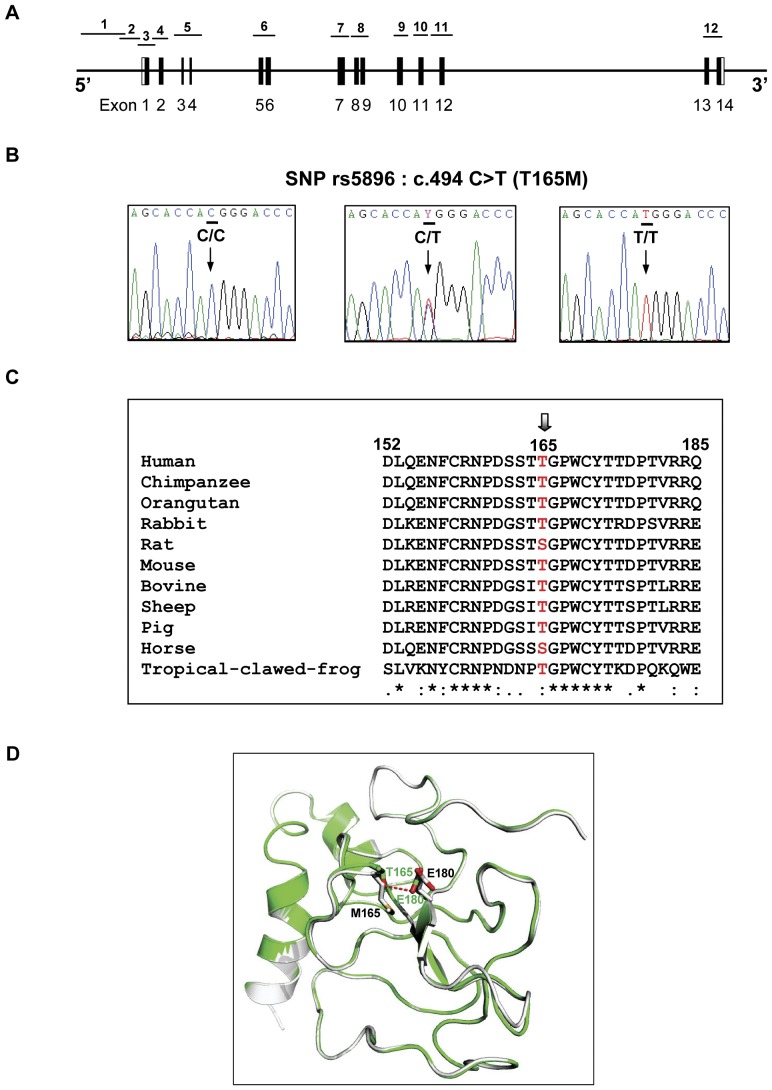
*F2* gene structure, SNP rs5896, alignment of prothrombin amino acid sequences, and 3D structure of UPTF1. A: Gene structure of *F2*. Exons 1–14 and intervening sequences (introns) are indicated by boxes and line, respectively. Fragment numbers 1 to 12 represent expected PCR products. B: DNA sequencing profile showing a SNP (rs5896, c.494 C>T) in exon 6, resulting in a substitution of threonine (T) by methionine (M) at position 165 (T165M). Vertical arrows indicate the positions of nucleotide variations. SNP genotypes are indicated by bold capital letters above the vertical arrows. C: Multiple alignment of amino acid sequences in a highly conserved region of prothrombin (F2), residues 152–185 (human sequence numbering) from eleven species. The symbols “*” and “:” under the alignment indicate the positions with conserved and conservative-changed amino acids, respectively. The T165 position is indicated by an arrow. D: Three dimensional (3D) structure of urinary prothrombin fragment 1 (UPTF1), showing an amino acid alteration, T165M. Wild-type T165 is indicated as green, which is superimposed by the altered amino acid, M165, indicated as gray. Wild-type residue is shown as green letters while the variant residue is presented as black letters. The dash line implies the predicted H-bond between T165 and E180 residues. The oxygen and sulfur atoms are shown with red and yellow, respectively.

All 14 exons, exon-intron boundaries, and promoter region of *F2* were amplified in 12 fragments and initially sequenced in 3 DNA samples each of the cases and control subjects (Figure 1A). A total of 6 SNPs, 1 exonic SNP (rs5896) and 5 intronic SNPs (rs2070850, rs2070852, rs1799867, rs2282687, and rs3136516), were detected (Figure 1B and Figure S1). All SNPs were analyzed for their functional impact by using four web-based programs (Mutation Taster, PolyPhen, VarioWatch, and SIFT). The 5 intronic SNPs were predicted as polymorphisms with no functional effect while the exonic SNP was predicted to be possibly damaging. The non-synonymous (ns) SNP, rs5896 (c.494 C>T), leading to a threonine (T) -to- methionine (M) substitution at amino acid 165 (T165M) was observed in exon 6 (Figure 1B). Multiple alignment of amino acid sequences of prothrombin from different species indicated that threonine 165 is in a highly conserved residue in the conserved region between residues 145 to 185 (Figure 1C).

### Genotyping of Single Nucleotide Polymorphism

**Figure 2:**
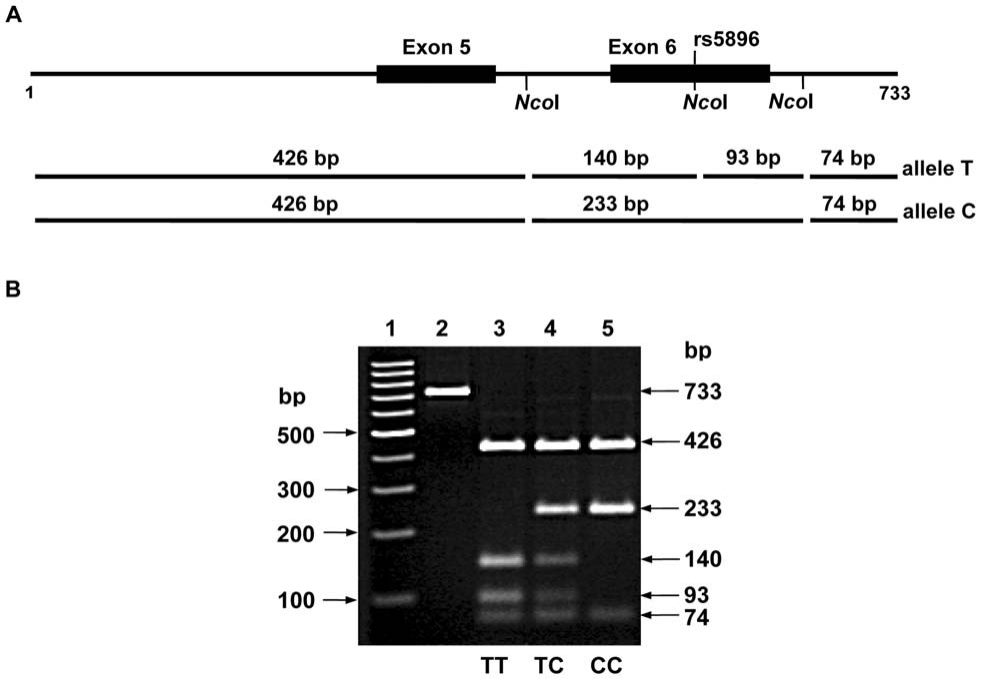
Analysis of rs5896 (c.494 C>T) by polymerase chain reaction - restriction fragment length polymorohism (PCR-RFLP). A: Locations of SNP rs5896 and restriction sites of *Nco*I on amplified DNA fragment (733 bp), containing exons 5 and 6. The expected RFLP patterns for genotyping of SNP rs5896 are indicated as lines with the lengths of 426, 140, 93 and 74 bp for allele T, and 426, 233 and 74 bp for allele C. B: *Nco*I-digested DNA patterns separated by electrophoresis on 3% agarose: lane 1, 100-bp ladder markers; lane 2, undigested PCR product (733 bp); lane 3, homozygote TT (426, 140, 93 and 74 bp); lane 4, heterozygote CT (426, 233, 140, 93 and 74 bp), and lane 5, homozygote CC (426, 233 and 74 bp).

Genotyping of *F2* polymorphism, rs5896 (c.494 C>T), was performed by PCR-RFLP method. The PCR product, 733 bp in size, was digested with restriction endonuclease – *Nco*I to generate 3 patterns, indicating 3 different genotypes: TT (426, 140, 93 and 74 bp), TC (426, 233, 140, 93 and 74 bp), and CC (426, 233, and 74 bp) (Figure 2). A total of 209 DNA samples of the patient group and 216 DNA samples of the control subject group were genotyped. The results showed that 86, 95 and 28 of the patients’ samples and 67, 101 and 48 of the control subjects’ samples were carrying the rs5896 genotypes TT, TC, and CC, respectively.

### Statistical Analysis of *F2* Polymorphisms and Kidney stone Disease

Analysis of genotype or allele frequencies of SNP rs5896 in the patients and control subjects using the web-based SNPStats program revealed their significant differences (Table 1). The model of inheritance considered by the least value of Akaike’s Information Criterion (AIC) was found to be recessive. The patients had significantly lower proportion of homozygous genotype of minor allele (CC) than the control subjects at *P* value of 0.017, indicating that CC was protective to the KSD with an odd ratio (OR) of 0.54, 95% CI 0.32–0.90. Likewise, the frequency of C allele in the patients was significantly lower than that of the control subjects (OR 0.68, 95% CI 0.51–0.89, *P* value = 0.005). When female and male groups of the patients and control subjects were separately analyzed, the genotype and allele frequencies in only the female group were significantly different at *P* values of 0.033 (OR 0.49, 95% CI 0.26–0.95) and 0.003 (OR 0.59, 95% CI 0.41–0.84), respectively (Table 2 and Table 3). Pair-wise linkage disequilibrium (LD) and haplotype block structure were estimated by Haploview 4.1 software, using the genotyping results of SNP rs5896 from this study with 10 other flanking SNPs (rs2070850, rs3136435, rs3136441, rs2070851, rs2080752, rs3136456, rs3136457, rs3136460, rs2282687, and rs3136516) from our previous study [10]. The SNP rs5896 was found in LD with other flanking SNPs. Interestingly, two haplotypes were dually associated with the kidney stone risk; one (TGCCGTCCGCG; rs5896 is underlined) with increased disease risk (*P* = 0.0060) and the other (CGTTCCCGCTA) with decreased disease risk (*P* = 0.0010). Furthermore, these two *F2* haplotypes were associated with increased and decreased disease risks in only the female group (*P* = 0.0092 and *P* = 0.0001, respectively) (Table S3–S5 and Figure S2).

**Table 1:** Genotype and allele frequencies of SNP rs5896 in *F2* in 209 patients with kidney stone disease and 216 control subjects.

Genotype	Genotype frequency (%)		OR (95% CI)	*X* ^2^	*P*	Allele	Allele frequency (%)		OR (95% CI)	*X* ^2^	*P*
	Control	Patient					Control	Patient			
T/T, T/C	77.8	86.6	1	5.6	**0.017**	T	54.4	63.9	1	7.9	**0.005**
C/C	22.2	13.4	0.54 (0.32–0.90)			C	45.6	36.1	0.68 (0.51–0.89)		

CI = confidence interval; OR = odd ratio.

**Table 2:** Genotype and allele frequencies of SNP rs5896 in *F2* in female group (132 patients with kidney stone disease and 126 control subjects).

Genotype	Genotype frequency (%)		OR (95% CI)	*X* ^2^	*P*	Allele	Allele frequency (%)		OR (95% CI)	*X* ^2^	*P*
	Control	Patient					Control	Patient			
T/T, T/C	77.0	87.1	1	4.5	**0.033**	T	52.8	65.5	1	8.7	**0.003**
C/C	23.0	12.9	0.49 (0.26–0.95)			C	47.2	34.5	0.59 (0.41–0.84)		

CI = confidence interval; OR = odd ratio.

**Table 3:** Genotype and allele frequencies of SNP rs5896 in *F2* in male group (77 patients with kidney stone disease and 90 control subjects).

Genotype	Genotype frequency (%)		OR (95% CI)	*X* ^2^	*P*	Allele	Allele frequency (%)		OR (95% CI)	*X* ^2^	*P*
	Control	Patient					Control	Patient			
T/T, T/C	78.9	85.7	1	1.3	0.250	T	56.7	61.0	1	0.6	0.418
C/C	21.1	14.3	0.62 (0.28–1.41)			C	43.3	39.0	0.84 (0.54–1.29)		

CI = confidence interval; OR = odd ratio.

### Structural Analysis of Human Prothrombin Frangment 1

Human prothrombin fragment 1 homologue was constructed by alignment and building profile using SWISS-MODEL workspace. This program compares protein sequence to sequence databases and calculates the statistical significance of matches (low E value) and identity in percentages. Bovine prothrombin fragment 1 (PDB: 2pf1A) was selected as the most suitable template. The alignment of human and bovine prothrombin fragment 1 showed 33 from 121 amino-acid difference with E value 8×10^−53^, 80.9% of sequence identity without any gap. Three-dimensional structure and *in silico* mutagenesis was studied. The effect of amino acid change on prothrombin fragment 1 structure was investigated by PyMOL VERSION 0.99rc6 program. The constructed human prothrombin fragment 1 of wild-type (threonine 165) and variant (methionine 165) and their alterations of H-bond forming patterns with glutamic acid 180 are shown in Figure 1D. Threonine is a polar, uncharged amino acid which contained hydroxyl- and methyl group as side chains. Hydroxyl group of threonine represents a strong electron donor while glutamic acid, a polar and acidic amino acid, acts as a strong electron acceptor. Thus, a hydrogen bond can be formed between the hydroxyl group of threonine 165 and the carboxylic group of glutamic acid 180. In contrast, methionine is a neutral, non-polar, hydrophobic amino acid. Methionine more favorably interacts with other methionine or hydrophobic amino acids. Thus, methionine 165 may be unable to form a hydrogen (H) bond with glutamic acid 180.

## Discussion

Kidney stone disease (KSD) is endemic in the Northeastern (NE) Thai population, causing major medical problems and economic burdens to the patients’ families and local communities. Our group has recently studied genetic polymorphisms of *F2*, encoding urinary prothrombin fragment 1 (UPTF1) – a stone inhibitor protein, and reported the association between *F2* haplotypes and genetic susceptibility or protection to the KSD in this population [10]. In the present study, we attempted to identify genetic variation in *F2* by sequencing in its promoter and all coding regions. One exonic non-synonymous SNP rs5896 (c.494 C>T, T165M), was found to potentially play a role in the KSD in the studied population.

We analyzed rs5896 in 209 patients and 216 control subjects by PCR-RFLP method. The genotype and allele frequencies between two groups were significantly different. The patients had significantly lower proportion of CC genotype and C allele than the control subjects (*P = *0.017 and 0.005). However, when we divided the data to analyze on the basis of their genders, significant differences were observed only in the female group with *P = *0.033 and 0.003 for genotype and allele frequencies, respectively. Two haplotypes were also found to be associated with increased and decreased disease risks in only the female group. These results indicate that rs5896 plays some functional role in KSD in the female group. The reason why rs5896 plays a role only in the female group is still unclear. Our previous data on the prevalence of KSD in the NE Thai population has shown a male predominance, with a male to female ratio of about 2∶1 [5]. Gender and sex-hormone genes are known to have some influence on the prevalence of KSD. In terms of complex genetic etiology of KSD, *F2* seems to be a modifying gene of the disease [10]. Thus, it is possible that different modifiers may play role in two different genders or this may reflex the complex interaction between *F2* and specific sex-hormone genes (e.g. testosterone or estrogen gene) in the male or female group. The relationship between SNP rs5896 and some relevant biochemical parameters of urine and serum was analyzed in a limited number of patients with available biochemical data; however, none of the analyzed parameters reached the significance level (p>0.05). With respect to this low statistical power, our finding needs to be replicated in larger sample sizes to validate this relationship. The relationships between SNP rs5896 and stone frequency or recurrence were not analyzed because they required a long- term follow up of the patients with KSD, which is not possible in the set up of our study.

The exonic location and non-synonymous nature of *F2* SNP rs5896 suggest that it may exert some effect on the UPTF1 function. The rs5896 C>T results in a substitution of threonine, an amino acid with uncharged and polar side chain, by methionine, an amino acid with non-polar side chain, at the position 165 (T165M) in the kringle 1 domain of UPTF1. Homologous modeling and *in silico* mutagenesis of T165M substitution revealed an alteration of H-bond forming pattern (Figure 1D). In contrast to threonine, methionine 165 is unable to form H-bond with glutamic acid 180. Although the impact of T165M substitution has not yet been reported, this change in H-bond forming is likely to consequently alter protein structure and function. The UPTF1 kringle 1 domain is important in protein-protein interactions with blood coagulation factors and it is believed to play a role in binding mediators (e.g. membranes, other proteins or phospholipids), and in regulating proteolytic activity [18]–[20].

UPTF1 is highly glycosylated and its carbohydrate moiety seems to influence functionality. Molecular modeling located two N-glycan sites (Asn78 and Asn100) and two O-glycan sites (Thr121 and Thr122) on the kringle domain of UPTF1 [21]. The relationship between UPTF1 glycosylation and CaOx stone pathogenesis was studied by evaluation of N-linked and O-linked oligosaccharides released from UPTF1, isolated from the urine of stone formers and non-stone formers. Different levels of sialylation were observed, suggesting a protective role of glycans [21]. The glycans on UPTF1 was reported to play a pivotal role in the protein ability to retard CaOx crystallization by the later study [22]. Although glycosylation is a post-translational modification of protein, genetic factors may well have contribution. In this regard, the T165M substitution (the same position as Thr122 in Webber *et al.* 2006 [21]) may affect UPTF1 glycosylation by destroying one O-glycan site. We propose that the minor (C) allele of SNP rs5896 (encoding threonine) – a protective allele for KSD, which is significantly more frequent in the control group than in the patient group, conserves the glycan site of UPTF1 and influences the UPTF1 inhibitory activity function in protection against CaOx crystallization. *F2* mRNA was found to significantly reduce in kidney of a hyperoxaluric rat model of nephrolithiasis [23] and many features of UPTF1 function have been studied [21], [22], [24], [25]. However, the precise mechanism of KSD with respect to UPTF1 remains unclear. Further study on the role of UPTF1 in kidney stone formation is still required.

Genetic contribution to KSD has been extensively recognized and variations of certain genes including osteopontin (*OPN*), vitamin D receptor (*VDR*), and calcium-sensing receptor (*CASR*) have been reported by candidate gene association studies. However, the contribution and pathogenic weight of these genes remains unclear [26]. Although the polymorphisms of candidate genes were examined in the patients with KSD, novel genetic variants and loci identified by genome-wide association studies (GWAS) have also been reported [27]–[30]. In addition, the current next-generation sequencing techniques that are feasible to sequence all exons or the whole genomes are likely to become the commonly used tool for identification of disease gene in the near future [31]. Our group is employing genome-wide linkage analysis and exome sequencing for identification of disease-causing genes and mutations in the NE Thai families with KSD.

In conclusion, the results of our study indicate the association between specific *F2* variation (T165M) and the risk of KSD in the NE Thai female patients, highlighting the role of UPTF1 in the kidney stone formation. Functional study of UPTF1 T165M should be further carried out to examine the link between genetic variation and biological function of the variant protein.

## Supporting Information

Figure S1 **Nucleotide sequencing profiles of 5 intronic SNPs of** ***F2*** **.** A-E: Portions of DNA sequencing profiles of *F2* from DNA samples of patients with kidney stone disease and control subjects showing SNP rs2070850 (c.240+83 C>T), SNP rs2070852 (c.423-7 G>C), rs1799867 (c.875-69 T>C), rs282687 (c.1654+290 T>C), and rs3136516 (c.1726-59 G>A), respectively. Vertical arrows indicate nucleotide variations. SNP genotypes are indicated by bold capital letters above the vertical arrows.
(DOC)

Figure S2 **Linkage disequilibrium (LD) plot.** LD plot showing D’ and LD block of 11 genotyped SNPs, including rs5896, in *F2* from 209 patients and 216 controls determined by the Haploview program. Genomic structure of *F2* and location of SNPs are indicated above the LD plot. Exons are indicated by black boxes and untranslated regions are represented in white. LD block is indicated by the black pentagon line. Squares represent LD and LOD score between SNPs. Numbers in boxes represent D’ (x 100). Bottom left panel displays the frequency of haplotype. The strength of LD is indicated with the bottom right-color scheme.
(DOC)

Table S1 **Characteristics of patients with kidney stone disease and control subjects.**
(DOC)

Table S2 **PCR primers for amplifications of** ***F2*** **in the regions of promoter and all exons.**
(DOC)

Table S3 **Analysis of association between** ***F2*** **haplotypes (constructed from SNP rs5896 plus 10 other SNPs) and kidney stone risk in combined female and male groups.**
(DOC)

Table S4 **Analysis of association between** ***F2*** **haplotypes (constructed from SNP rs5896 plus 10 other SNPs) and kidney stone risk in female group.**
(DOC)

Table S5 **Analysis of association between** ***F2*** **haplotypes (constructed from SNP rs5896 plus 10 other SNPs) and kidney stone risk in male group.**
(DOC)

## References

[1] Aegukkatajit S, Nagaphant A, Nuhung R, Sinturat R, Nugoonsawat P, et al (1994) Epidemiological study of urinary stones based on operative theater data at regional hospitals and general hospitals of public health region-5, Thailand. J Med Assoc Thai 77: 484–487. 7706968

[2] Yanagawa M, Kawamura J, Onishi T, Soga N, Kameda K, et al (1997) Incidence of urolithiasis in northeast Thailand. Int J Urol 4: 537–540. 9477179 10.1111/j.1442-2042.1997.tb00304.x

[3] Nilwarangkur S, Malasit P, Nimmannit S, Susaengrat W, Ong-Aj-Yooth S, et al (1990) Urinary constituents in an endemic area of stones and renal tubular acidosis in northeastern Thailand. Southeast Asian J Trop Med Public Health 21: 437–441. 2075484

[4] Sriboonlue P, Prasongwattana V, Tungsanga K, Tosukhowong P, Phantumvanit P, et al (1991) Blood and urinary aggregator and inhibitor composition in controls and renal-stone patients from northeastern Thailand. Nephron 59: 591–596. 1766498 10.1159/000186649

[5] Sritippayawan S, Borwornpadungkitti S, Paemanee A, Predanon C, Susaengrat W, et al (2009) Evidence suggesting a genetic contribution to kidney stone in Northeastern Thai population. Urol Res 37: 141–146. 19387627 10.1007/s00240-009-0189-1

[6] Howard JE, Thomas WC (1959) Some observations on rachitic rat cartilage of probable significance in the etiology of renal calculi. Trans Am Clin Climatol Assoc 70: 94–102. 21407783 PMC2249124

[7] Ryall RL (1997) Urinary inhibitors of calcium oxalate crystallization and their potential role in stone formation. World J Urol 15: 155–164. 9228722 10.1007/BF02201852

[8] Khan SR, Kok DJ (2004) Modulators of urinary stone formation. Front Biosci 9: 1450–1482. 14977559 10.2741/1347

[9] Basavaraj DR, Biyani CS, Browning AJ, Cartledg JJ (2007) The role of urinary kidney stone inhibitors and promoters in the pathogenesis of calcium containing renal stones. EAU-EBU Update Series 5: 126–136.

[10] Rungroj N, Sritippayawan S, Thongnoppakhun W, Paemanee A, Sawasdee N, et al (2011) Prothrombin haplotype associated with kidney stone disease in Northeastern Thai patients. Urology 77: 249.e17–23. 10.1016/j.urology.2010.07.494 21067798

[11] Royle NJ, Irwin DM, Koschinsky ML, MacGillivray RT, Hamerton JL (1987) Human genes encoding prothrombin and ceruloplasmin map to 11p11-q12 and 3q21–24, respectively. Somat Cell Mol Genet 13: 285–292. 3474786 10.1007/BF01535211

[12] Degen SJ, Davie EW (1987) Nucleotide sequence of the gene for human prothrombin. Biochemistry 26: 6165–6177. 2825773 10.1021/bi00393a033

[13] Stapleton AM, Ryall RL (1995) Blood coagulation proteins and urolithiasis are linked: crystal matrix protein is the F1 activation peptide of human prothrombin. Br J Urol 75: 712–719. 7613825 10.1111/j.1464-410x.1995.tb07377.x

[14] Doyle IR, Ryall RL, Marshall VR (1991) Inclusion of proteins into calcium oxalate crystals precipitated from human urine: a highly selective phenomenon. Clin Chem 37: 1589–1594. 1893595

[15] Mann KG (1976) Prothrombin. Methods Enzymol 45: 123–156. 1011988 10.1016/s0076-6879(76)45016-4

[16] Ryall RL, Grover PK, Stapleton AM, Barrell DK, Tang Y, et al (1995) The urinary F1 activation peptide of human prothrombin is a potent inhibitor of calcium oxalate crystallization in undiluted human urine *in vitro.* . Clin Sci (Lond) 89: 533–541. 8549069 10.1042/cs0890533

[17] Grover PK, Moritz RL, Simpson RJ, Ryall RL (1998) Inhibition of growth and aggregation of calcium oxalate crystals *in vitro*: a comparison of four human proteins. Eur J Biochem 253: 637–644. 9654060 10.1046/j.1432-1327.1998.2530637.x

[18] Fujikawa K, McMullen BA (1985) Amino acid sequence of the heavy chain of human alpha-factor XIIa (activated Hageman factor). J Biol Chem 260: 5328–5341. 3886654

[19] Patthy L, Trexler M, Váli Z, Bányai L, Váradi A (1984) Kringles: modules specialized for protein binding. Homology of the gelatin-binding region of fibronectin with the kringle structures of proteases. FEBS Lett 171: 131–136. 6373375 10.1016/0014-5793(84)80473-1

[20] Atkinson RA, Williams RJ (1990) Solution structure of the kringle 4 domain from human plasminogen by 1H nuclear magnetic resonance spectroscopy and distance geometry. J Mol Biol 212: 541–552. 2157850 10.1016/0022-2836(90)90330-O

[21] Webber D, Radcliffe CM, Royle L, Tobiasen G, Merry AH, et al (2006) Sialylation of urinary prothrombin fragment 1 is implicated as a contributory factor in the risk of calcium oxalate kidney stone formation. FEBS J 273: 3024–3037. 16817853 10.1111/j.1742-4658.2006.05314.x

[22] Webber D, Rodgers AL, Sturrock ED (2007) Glycosylation of prothrombin fragment 1 governs calcium oxalate crystal nucleation and aggregation, but not crystal growth. Urol Res 35: 277–285. 17987287 10.1007/s00240-007-0119-z

[23] Grover PK, Miyazawa K, Coleman M, Stahl J, Ryall RL (2006) Renal prothrombin mRNA is significantly decreased in a hyperoxaluric rat model of nephrolithiasis. J Pathol 210: 273–281. 16981243 10.1002/path.2061

[24] Liu J, Chen J, Wang T, Wang S, Ye Z (2005) Effects of urinary prothrombin fragment 1 in the formation of calcium oxalate calculus. J Urol 173: 113–116. 15592049 10.1097/01.ju.0000146847.24571.c8

[25] Liu J, Wang T, Chen J, Wang S, Ye Z (2006) Decreased inhibitory activity of prothrombin to calcium oxalate crystallization by specific chemical modification of its gamma-carboxyglutamic acid residues. Urology 67: 201–203. 16413375 10.1016/j.urology.2005.07.058

[26] Vezzoli G, Terranegra A, Arcidiacono T, Soldati L (2011) Genetics and calcium nephrolithiasis. Kidney Int 80: 587–593. 20962745 10.1038/ki.2010.430

[27] Thorleifsson G, Holm H, Edvardsson V, Walters GB, Styrkarsdottir U, et al (2009) Sequence variants in the CLDN14 gene associate with kidney stones and bone mineral density. Nat Genet 41: 926–930. 19561606 10.1038/ng.404

[28] Gudbjartsson DF, Holm H, Indridason OS, Thorleifsson G, Edvardsson V, et al (2010) Association of variants at UMOD with chronic kidney disease and kidney stones-role of age and comorbid diseases. PLoS Genet 6: e1001039. 20686651 10.1371/journal.pgen.1001039 PMC2912386

[29] Tore S, Casula S, Casu G, Concas MP, Pistidda P, et al (2011) Application of a new method for GWAS in a related case/control sample with known pedigree structure: identification of new loci for nephrolithiasis. PLoS Genet 7: e1001281. 21283782 10.1371/journal.pgen.1001281 PMC3024262

[30] Urabe Y, Tanikawa C, Takahashi A, Okada Y, Morizono T, et al (2012) A genome-wide association study of nephrolithiasis in the Japanese population identifies novel susceptible Loci at 5q35.3, 7p14.3, and 13q14.1. PLoS Genet 8: e1002541. 22396660 10.1371/journal.pgen.1002541 PMC3291538

[31] Gilissen C, Hoischen A, Brunner HG, Veltman JA (2012) Disease gene identification strategies for exome sequencing. Eur J Hum Genet 20: 490–497. 22258526 10.1038/ejhg.2011.258 PMC3330229

