# Supplementary material for: Association between Human Prothrombin Variant (T165M) and Kidney Stone Disease
Source: PLoS One. 2012 Sep 19;7(9):e45533. doi: 10.1371/journal.pone.0045533 (PMC3446884; doi:10.1371/journal.pone.0045533)
Supplement: Figure S1 — Nucleotide sequencing profiles of 5 intronic SNPs of F2 . A-E: Portions of DNA sequencing profiles of F2 from DNA samples of patients with kidney stone disease and control subjects showing SNP rs2070850 (c.240+83 C>T), SNP rs2070852 (c.423-7 G>C), rs1799867 (c.875-69 T>C), rs282687 (c.1654+290 T>C), and rs3136516 (c.1726-59 G>A), respectively. Vertical arrows indicate nucleotide variations. SNP genotypes are indicated by bold capital letters above the vertical arrows. (DOC) [file pone.0045533.s001.doc]

**Figure S1**

Nucleotide sequencing profiles of 5 intronic SNPs of *F2*

**
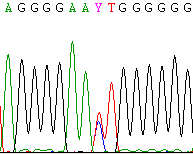

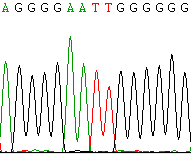

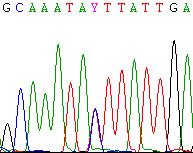

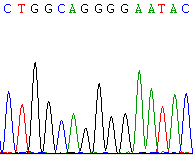

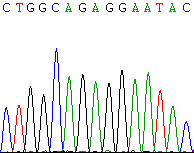

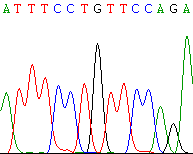

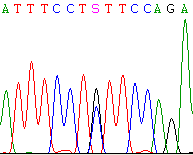

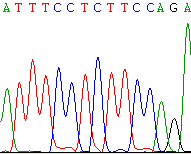

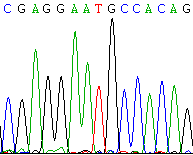

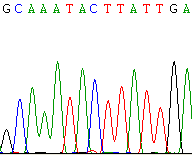
**


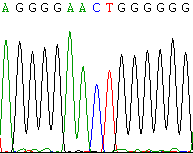


**C/C**


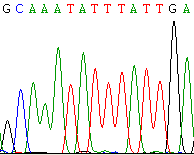


**T/T**


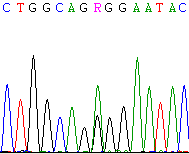


**G/A**


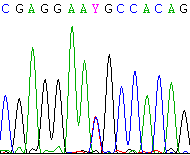


**C/T**


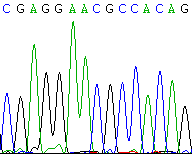


**C/C**

**T/C**

**T/T**

**T/C**

**G/G**

**A/A**

**G/G**

**G/C**

**C/C**

**T/T**

**A** SNP rs2070850 : c.240+83 C>T

**C/C**

**B** SNP rs2070852 : c.423-7 G>C

**C** SNP rs1799867 : c.875-69 T>C

**D** SNP rs2282687 : c.1654+290 T>C

**E** SNP rs3136516 : c.1726-59 G>A
