# Supplementary figures and images for: Association between Human Prothrombin Variant (T165M) and Kidney Stone Disease
Source: PLoS One. 2012 Sep 19;7(9):e45533. doi: 10.1371/journal.pone.0045533 (PMC3446884; doi:10.1371/journal.pone.0045533)

**Figure S2**

Linkage disequilibrium (LD) plot


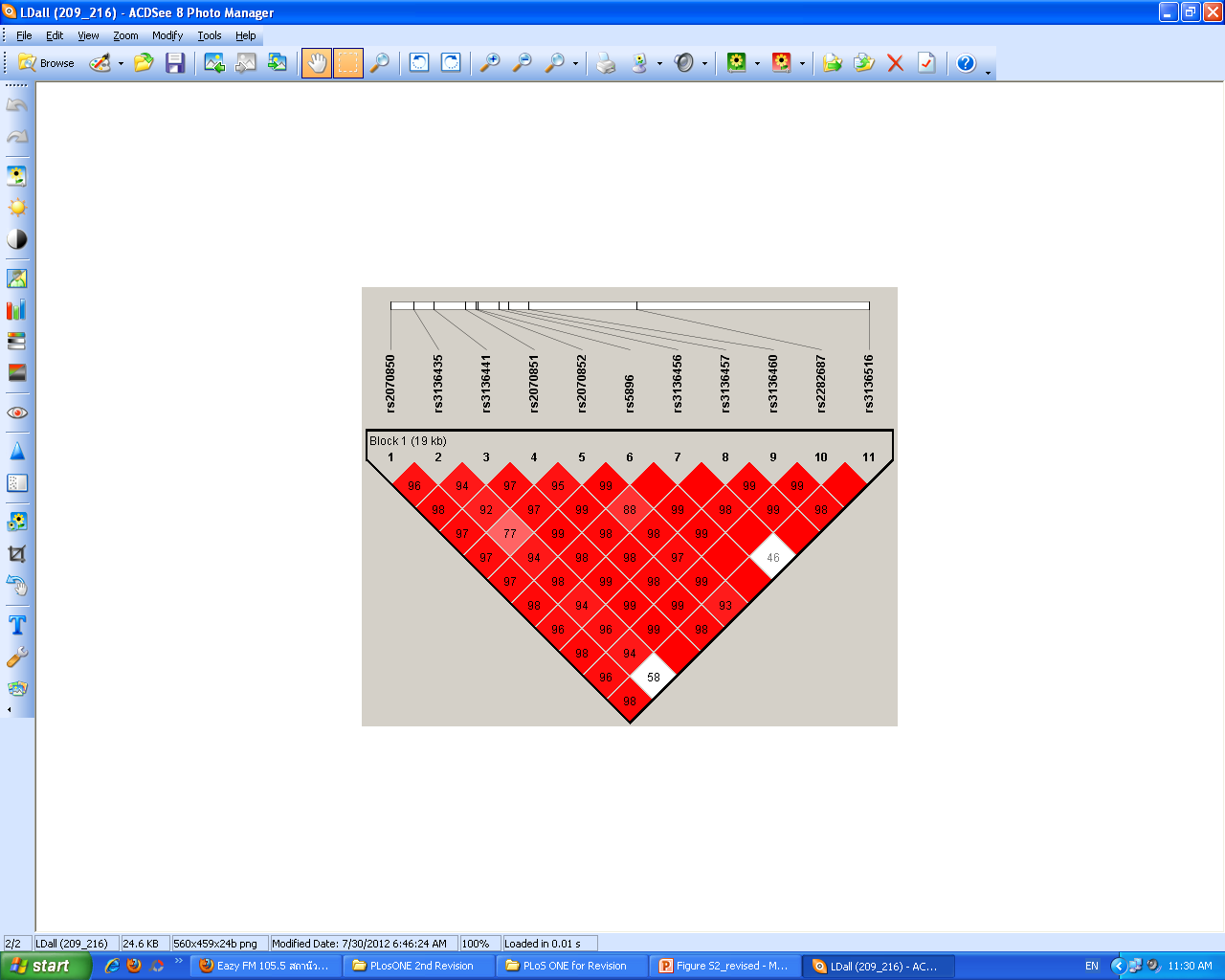

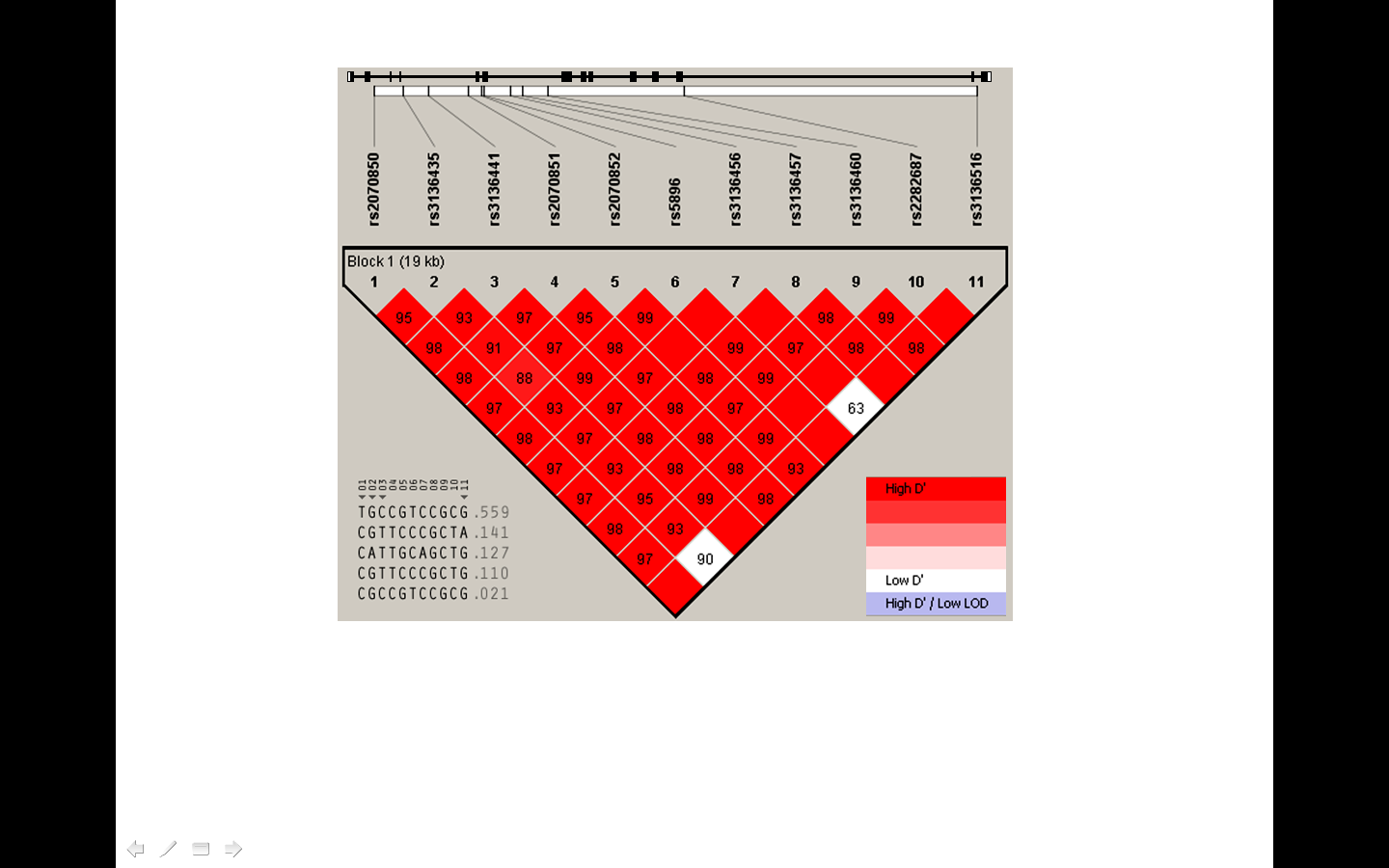

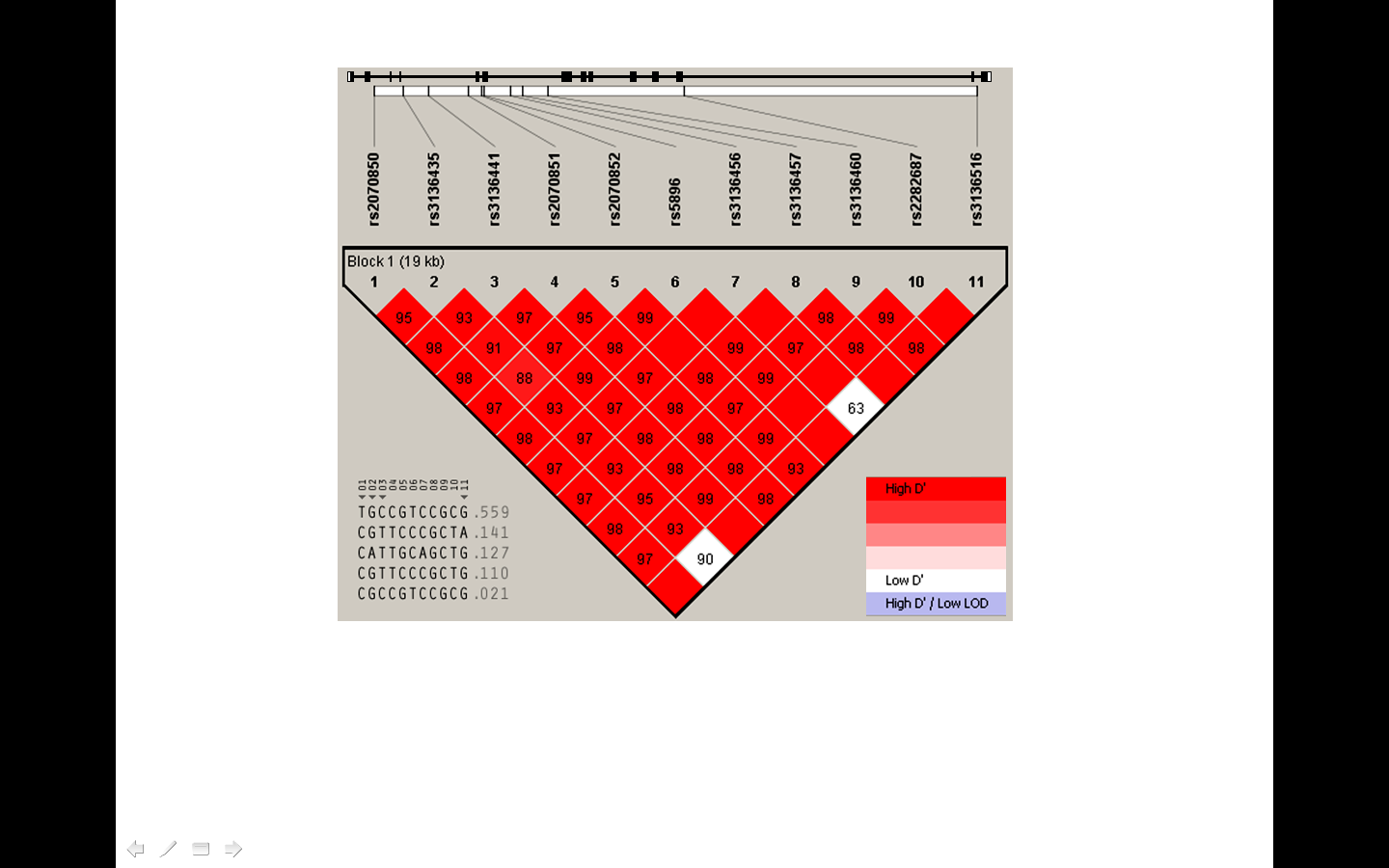

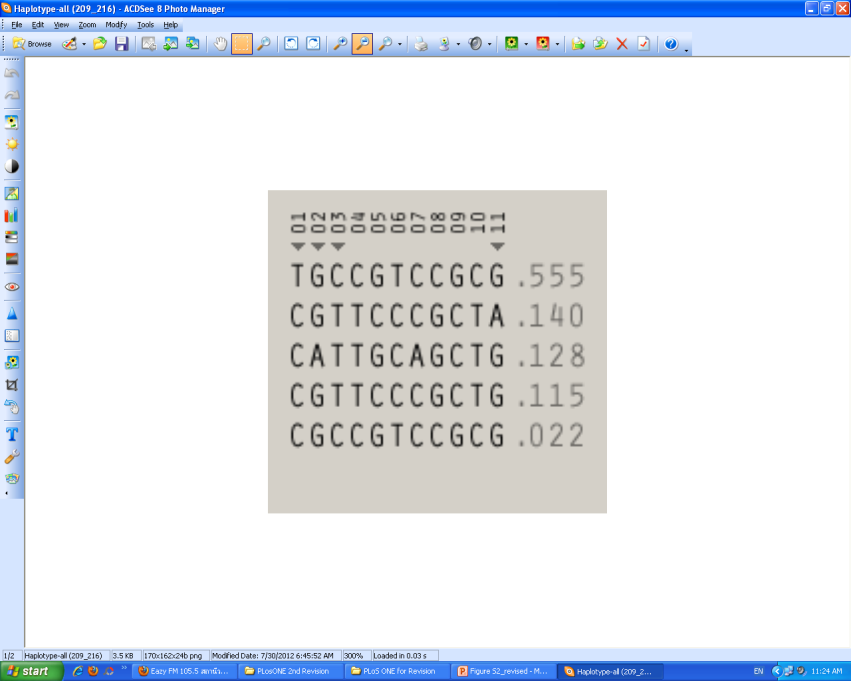

Supplement: Figure S2 — Linkage disequilibrium (LD) plot. LD plot showing D’ and LD block of 11 genotyped SNPs, including rs5896, in F2 from 209 patients and 216 controls determined by the Haploview program. Genomic structure of F2 and location of SNPs are indicated above the LD plot. Exons are indicated by black boxes and untranslated regions are represented in white. LD block is indicated by the black pentagon line. Squares represent LD and LOD score between SNPs. Numbers in boxes represent D’ (x 100). Bottom left panel displays the frequency of haplotype. The strength of LD is indicated with the bottom right-color scheme. (DOC) [file pone.0045533.s002.doc]
