# Supplementary material for: Association between Human Prothrombin Variant (T165M) and Kidney Stone Disease
Source: PLoS One. 2012 Sep 19;7(9):e45533. doi: 10.1371/journal.pone.0045533 (PMC3446884; doi:10.1371/journal.pone.0045533)
Supplement: Table S1 — Characteristics of patients with kidney stone disease and control subjects. (DOC) [file pone.0045533.s003.doc]

**Table S1.** Characteristics of patients with kidney stone disease and control subjects.

| **Characteristics** | **Patients (n)** | **%** | **Controls (n)** | **%** |
| --- | --- | --- | --- | --- |
| **Gender** |  |  |  |  |
| Female | 135 | 62.50 | 126 | 58.33 |
| Male | 81 | 37.50 | 90 | 41.67 |
| **Age** |  |  |  |  |
| Mean (years) | 49.7 ±11.7 | - | 50.5 ±13.5 | - |
| Range (years) | 22-80 | - | 22-84 | - |
| **Familial history** |  |  |  |  |
| Yes | 139 | 64.35 | - | - |
| No | 77 | 35.65 | - | - |
| **Stone number** |  |  |  |  |
| Single | 49 | 22.68 | - | - |
| Multiple | 104 | 48.15 | - | - |
| Unknown | 63 | 29.17 | - | - |
| **Position of stone** |  |  |  |  |
| Kidney | 181 | 83.80 | - | - |
| Ureter | 13 | 6.02 | - | - |
| Kidney and ureter | 20 | 9.26 | - | - |
| Kidney and bladder | 2 | 0.92 | - | - |
| **Main stone type** |  |  |  |  |
| Whewellite | 39 | 18.05 | - | - |
| Dahllite | 36 | 16.67 | - | - |
| Weddellite | 4 | 1.85 | - | - |
| Uric acid | 5 | 2.31 | - | - |
| Struvite | 2 | 0.93 | - | - |
| Unknown (not determined) | 130 | 60.19 | - | - |
