# Supplementary material for: Association between Human Prothrombin Variant (T165M) and Kidney Stone Disease
Source: PLoS One. 2012 Sep 19;7(9):e45533. doi: 10.1371/journal.pone.0045533 (PMC3446884; doi:10.1371/journal.pone.0045533)
Supplement: Table S2 — PCR primers for amplifications of F2 in the regions of promoter and all exons. (DOC) [file pone.0045533.s004.doc]

**Table S2.** PCR primers for amplifications of *F2* in the regions of promoter and all exons.

| Product | Primer name | Sequencing (5’🡪3’) | Product size (bp) |
| --- | --- | --- | --- |
| Promoter 1 | F2_proL1 | CGCCTGTAATCCCCACTTTG | 1,533 |
|  | F2_proR1 | ATGGGATGCACCAGGGATAG |  |
| Promoter 2 | F2_LF1 | AGTCCCTGGACCTGACTCCT | 673 |
|  | F2ProR2 | TTGTCTGTCCTGACCCCTCT |  |
| Exon 1 | F2Ex1L | TAGACCATCCATCCCTGCTC | 571 |
|  | F2Ex1R | TGGCTTCTGAGAGAGGCTGT |  |
| Exon 2 | F2Ex2L | TGGAAAGAGAATGGCTGCTT | 513 |
|  | F2Ex2R | TGAAATGAGGCTGTGAGCAG |  |
| Exon 3-4 | F2Ex3L | GTGTCGCCTTTCCTGTCTGTAG | 924 |
|  | F2R2.1 | GAGGCAGAGAATTGCTTGAA |  |
| Exon 5-6 | F2F4 | GCCAAGAGAAGCCACAGAAT | 733 |
|  | F2LR1 | TGCCTGGGTAGCCAGTATTC |  |
| Exon 7 | F2Ex7L | GTCACACAGGCAGAAAGCAG | 593 |
|  | F2Ex7R | GGGCCTCAGTGTACTTATCTGTG |  |
| Exon 8-9 | F2Ex8-9L | ACGCTTAACCTCTGCACCAA | 577 |
|  | F2Ex8-9R | ATCCTGGATGCTCAGCTGTT |  |
| Exon 10 | F2Ex10L | GGGATTGTTACTTCTAGGGCTGG | 458 |
|  | F2Ex10R | GGAGGGACCAGGAGACAGACG |  |
| Exon 11 | F2Ex11L | CCAGGCTGTCTGACTCCAAAG | 494 |
|  | F2Ex11R | ACTCCAAGAAACCGAGGCC |  |
| Exon 12 | F2Ex12L | CAGCTCTGGCGTTTTAGATTC | 715 |
|  | F2R7 | CTGGTCAGGGATTGCTGTTT |  |
| Exon 13-14 | F2F8 | GGGTAAGTGGACTCTCACCAG | 619 |
|  | F2Ex13-14R | AGTAGTATTACTGGCTCTTCCTGAG |  |
