# Supplementary material for: Association between Human Prothrombin Variant (T165M) and Kidney Stone Disease
Source: PLoS One. 2012 Sep 19;7(9):e45533. doi: 10.1371/journal.pone.0045533 (PMC3446884; doi:10.1371/journal.pone.0045533)
Supplement: Table S3 — Analysis of association between F2 haplotypes (constructed from SNP rs5896 plus 10 other SNPs) and kidney stone risk in combined female and male groups. (DOC) [file pone.0045533.s005.doc]

**Table S3.** Analysis of association between *F2* haplotypes (constructed from SNP rs5896 plus 10 other SNPs) and kidney stone risk in combined female and male groups.

| Haplotype | Frequency of haplotype | | OR (95% CI) | χ2 | *P* |
| --- | --- | --- | --- | --- | --- |
|  | Control (n = 216) | Patient (n = 209) |  |  |  |
| TGCCGTCCGCG | 0.509 | 0.603 | 1.464 (1.115-1.922) | 7.565 | **0.0060** |
| CGTTCCCGCTA | 0.178 | 0.100 | 0.514 (0.343-0.769) | 10.739 | **0.0010** |
| CATTGCAGCTG | 0.136 | 0.119 | 0.853 (0.569-1.278) | 0.598 | 0.4393 |
| CGTTCCCGCTG | 0.121 | 0.109 | 0.891 (0.584-1.359) | 0.283 | 0.5948 |
| CGCCGTCCGCG | 0.019 | 0.026 | 1.414 (0.565-3.541) | 0.569 | 0.4508 |

Order of 11 SNPs in haplotypes; rs2070850, rs3136435, rs3136441, rs2070851, rs2080752, rs5896, rs3136456, rs3136457, rs3136460, rs2282687, and rs3136516.

SNP rs5896 is underlined in the haplotype.

CI = confidence interval; OR = odds ratio.
