# Supplementary material for: Association between Human Prothrombin Variant (T165M) and Kidney Stone Disease
Source: PLoS One. 2012 Sep 19;7(9):e45533. doi: 10.1371/journal.pone.0045533 (PMC3446884; doi:10.1371/journal.pone.0045533)
Supplement: Table S4 — Analysis of association between F2 haplotypes (constructed from SNP rs5896 plus 10 other SNPs) and kidney stone risk in female group. (DOC) [file pone.0045533.s006.doc]

**Table S4.** Analysis of association between *F2* haplotypes (constructed from SNP rs5896 plus 10 other SNPs) and kidney stone risk in female group.

| Haplotype | Frequency of haplotype | | OR (95% CI) | χ2 | *P* |
| --- | --- | --- | --- | --- | --- |
|  | Control (n = 126) | Patient (n = 132) |  |  |  |
| TGCCGTCCGCG | 0.496 | 0.610 | 1.591 (1.121-2.256) | 6.783 | **0.0092** |
| CGTTCCCGCTA | 0.226 | 0.102 | 0.389 (0.237-0.639) | 14.524 | **0.0001** |
| CATTGCAGCTG | 0.111 | 0.113 | 1.026 (0.593-1.774) | 0.009 | 0.9243 |
| CGTTCCCGCTG | 0.119 | 0.091 | 0.741 (0.421-1.304) | 1.095 | 0.2953 |
| CGCCGTCCGCG | 0.020 | 0.020 | 1.513 (0.492-4.657) | 0.550 | 0.4583 |

Order of 11 SNPs in haplotypes; rs2070850, rs3136435, rs3136441, rs2070851, rs2080752, rs5896, rs3136456, rs3136457, rs3136460, rs2282687, and rs3136516.

SNP rs5896 is underlined in the haplotype.

CI = confidence interval; OR = odds ratio.
