# Supplementary material for: Association between Human Prothrombin Variant (T165M) and Kidney Stone Disease
Source: PLoS One. 2012 Sep 19;7(9):e45533. doi: 10.1371/journal.pone.0045533 (PMC3446884; doi:10.1371/journal.pone.0045533)
Supplement: Table S5 — Analysis of association between F2 haplotypes (constructed from SNP rs5896 plus 10 other SNPs) and kidney stone risk in male group. (DOC) [file pone.0045533.s007.doc]

**Table S5.** Analysis of association between *F2* haplotypes (constructed from SNP rs5896 plus 10 other SNPs) and kidney stone risk in male group.

| Haplotype | Frequency of haplotype | | OR (95% CI) | χ2 | *P* |
| --- | --- | --- | --- | --- | --- |
|  | Control (n = 90) | Patient (n = 77) |  |  |  |
| TGCCGTCCGCG | 0.522 | 0.591 | 1.322 (0.856-2.041) | 1.583 | 0.2083 |
| CGTTCCCGCTA | 0.116 | 0.110 | 0.940 (0.476-1.853) | 0.031 | 0.8607 |
| CATTGCAGCTG | 0.172 | 0.123 | 0.676 (0.365-1.253) | 1.556 | 0.2123 |
| CGTTCCCGCTG | 0.122 | 0.130 | 1.072 (0.561-2.049) | 0.046 | 0.8307 |
| CGCCGTCCGCG | 0.017 | 0.020 | 1.172 (0.233-5.893) | 0.037 | 0.8476 |

Order of 11 SNPs in haplotypes; rs2070850, rs3136435, rs3136441, rs2070851, rs2080752, rs5896, rs3136456, rs3136457, rs3136460, rs2282687, and rs3136516.

SNP rs5896 is underlined in the haplotype.

CI = confidence interval; OR = odds ratio.
